# Biochemical and Microbiological Aspects of the Oral Cavity of Children and Young People with Neurological Impairment and Oropharyngeal Dysphagia

**DOI:** 10.3390/life13061342

**Published:** 2023-06-08

**Authors:** Janaina Costa Marangon Duarte, Isabela Bazzo Costa, Daniel de Bortoli Teixeira, Luiz Fernando Fregatto, Claudemir Gregorio Mendes, Aline Maria Noli Mascarin, Salum Bueno da Silveira Junior, Bianca Eduarda Baptistella Mesquita Serva, Livia Picchi Comar, Roberta Gonçalves da Silva, Daniela Vieira Buchaim, Rogerio Leone Buchaim, Eduardo Federighi Baisi Chagas, Francisco Agostinho Junior, Paula Cristina Cola

**Affiliations:** 1 Postgraduate Program in Structural and Functional Interactions in Rehabilitation, University of Marilia (UNIMAR), Marilia 17525-902, Brazilefbchagas@unimar.br (E.F.B.C.); 2 Postgraduate Program in Animal Health, Production and Environment, University of Marilia (UNIMAR), Marilia 17525-902, Brazil; 3 Nursing School, University of Marilia (UNIMAR), Marilia 17525-902, Brazil; 4 UNIMAR Beneficent Hospital (HBU), University of Marilia (UNIMAR), Marilia 17525-160, Brazil; 5 Faculty of Pharmacy and Biomedicine, University of Marilia (UNIMAR), Marilia 17525-902, Brazil; 6 Dentistry School, University of Marilia (UNIMAR), Marilia 17525-902, Brazil; 7 Dysphagia Research Rehabilitation Center, Graduate of Speech, Language and Hearing Sciences Department, São Paulo State University (UNESP), Marilia 17525-900, Brazil; 8 Medical School, University Center of Adamantina (UNIFAI), Adamantina 17800-000, Brazil; 9 Department of Biological Sciences, Bauru School of Dentistry (FOB/USP), University of São Paulo, Bauru 17012-901, Brazil; rogerio@fob.usp.br; 10 Graduate Program in Anatomy of Domestic and Wild Animals, Faculty of Veterinary Medicine and Animal Science, University of São Paulo (FMVZ/USP), São Paulo 05508-270, Brazil; 11 Child’s Love Project, Projeto Amor de Criança, University of Marilia (UNIMAR), Marilia 17525-902, Brazil; 12 Speech Therapy Department, São Paulo State University (UNESP), Marilia 17525-900, Brazil

**Keywords:** dental calculus, deglutition disorder, oral hygiene, neurological disorders, saliva, dysphagia

## Abstract

The components and the salivary flow have a direct influence on the composition of the oral microbiota of children and young people with oropharyngeal dysphagia, and studies have already demonstrated the excessive accumulation of supragingival dental calculus in individuals with enteral nutrition. This study aimed to compare the oral hygiene, biochemical, and microbiological aspects of the oral cavity of children and young people with neurological impairment and oropharyngeal dysphagia. Forty children and young people with neurological impairment and oropharyngeal dysphagia were enrolled and divided into two groups: group I, encompassing 20 participants fed via gastrostomy; and group II, encompassing 20 participants fed via the oral route. Oral hygiene and salivary pH and flow were assessed, and a polymerase chain reaction was performed to evaluate the messenger RNA expressions of *Porphyromonas gingivalis*, *Tanerella forsythia*, and *Treponema denticola*. In groups I and II, the mean Oral Hygiene Index—Simplified scores were 4 and 2, respectively, showing a significant difference; the mean Calculus Index scores were 2 and 0, respectively, showing a significant difference; and the mean pH was 7.5 and 6.0, respectively, showing a significant difference. Bacterial analysis indicated no association between the two groups. It can be concluded that children and young people who use gastrostomy had a poorer oral hygiene, greater dental calculus deposition, and higher salivary pH. The saliva of patients in both groups contained *Porphyromonas gingivalis*, *Tanerella forsythia*, and *Treponema denticola*.

## 1. Introduction

Neurological impairments in children and young adults are usually accompanied by developmental changes, such as mental deficiency, attention deficit/hyperactivity disorder, dyslexia, and dysphagia [1]. The prevalence of neurogenic oropharyngeal dysphagia ranges from 43% to 99% in patients with cerebral palsy, causing nutritional and hydration losses, pulmonary complications, and food pleasure [2]. Gastrostomy, an alternative feeding route, causes weight gain, reverses or prevents malnutrition, reduces feeding time, facilitates medication administration, reduces respiratory problems and hospitalizations, and improves the caregivers’ quality of life [3,4].

Patients with neurological impairment often present with compromised oral care. Moreover, oral hygiene is maintained unsatisfactorily in these patients because of impaired motor coordination. Generally, patients with neurological impairment present with oral dysfunctions, such as pathological oral and primitive reflexes, drooling, xerostomia, and abnormal movements of the lips, tongue, and cheeks [5,6,7,8]. Children and young people with neurological impairment and oropharyngeal dysphagia who are fed via gastrostomy present with worse oral hygiene compared to those who are fed orally [9].

Impaired oral hygiene is due to bacterial plaque and dental calculus depositions in the permanent or deciduous dentition. The evidence thus far demonstrates that calculus deposition is greater in children and young people fed via gastrostomy. Patients with neurological impairment who are fed via gastrostomy have increased dental calculus formation [10,11,12,13]. 

Oropharyngeal dysphagia and poor oral hygiene can increase salivary pH and reduce salivary flow, predisposing patients to the colonization of *Klebsiella pneumoniae*, an enterobacteria causing pneumonia and hospital-acquired infections [14]. Adolescents with cerebral palsy have reduced salivary flow, which reduces the activity of enzymes, such as peroxidases, amylases, and total proteins in saliva, lowers the buffer capacity, and increases the sialic acid concentration, which are risk factors for oral diseases [15,16].

The microbiota involved in periodontal infections are considered to be mixed, as several species of microorganisms play specific roles in the etiopathogenesis of periodontal infections. Among these microorganisms, the Gram-negative anaerobes include *Prevotella, Fusobacterium*, *Porphyromonas*, *Pseudomonas*, *Aggregatibacter*, *Campylobacter*, and *Treponema*, particularly *Prevotella intermedia*, *Fusobacterium nucleatum*, *Porphyromonas gingivalis*, and *Aggregatibacter actinomycetemcomitans* [17,18]. *Porphyromonas gingivalis*, *Tannerella forsythia*, and *Treponema denticola* cause periodontal diseases and are prognostic factors for periodontitis [19,20]. 

The hypothesis this study was that children and young people with neurological impairment and oropharyngeal dysphagia fed via gastrostomy present with poorer oral hygiene, higher salivary pH, highest salivary flow, and greater oral bacterial colonization compared to those fed via the oral route.

The aim of the present study was to compare the oral hygiene, salivary pH and flow, and oral microbiota of patients with neurological impairment and oropharyngeal dysphagia fed through gastrostomy or the oral route. 

## 2. Materials and Methods

### 2.1. Study Type and Ethical Considerations

The design of this study was classified as cross-sectional and conducted according to the Strengthening the Reporting of Observational studies in Epidemiology (STROBE) [21]. The study protocol was approved by the Research Ethics Committee of the Institution University of Marilia (UNIMAR, approval number: 4.624.559; 31 March 2021). Informed consent was obtained from the participants or their guardians. 

### 2.2. Setting

The participants were selected from the outpatient department, and medical records were used to confirm the diagnosis of oropharyngeal dysphagia. Follow-ups were performed at the Specialties Clinic of Unimar Beneficent Hospital (HBU, UNIMAR, University of Marilia, Brazil) “Amor de Criança” project. Currently, the “Amor de Criança” project includes a population of 250 patients, mostly diagnosed with cerebral palsy and genetic syndromes, 50 of them with gastrostomy. The project has a multidisciplinary team that includes gastropediatricians, speech therapists, physiotherapists, occupational therapists, nurses, pharmacists, nutritionists, pediatric dentists, neuropediatricians, pulmonologists, orthopedists, and geneticists. The children included in the project are constantly monitored, and during the consultations, certain relevant aspects are evaluated, such as neuropsychomotor development, the degree of motor deficit, the presence of epilepsy requiring pharmacological treatment, swallowing, drug therapy, and scheduled return according to the needs of each patient.

### 2.3. Participant Selection 

#### Inclusion and Exclusion Criteria

In this clinical study, 40 individuals participated, including children, adolescents, and young adults, where 22 were male and 18 were female. The age of the participants was in the range of 2 to 22 years, with a mean of 8.5 years. In these study participants, all had neurological impairment (cerebral palsy or genetic syndrome), with neurogenic oropharyngeal dysphagia. They were divided into two groups, with group I consisting of 20 children and young people fed via gastrostomy and group II composed of 20 children and young people fed orally.

The minimum sample size was estimated at 17 sample elements per group to analyze the median difference between two independent groups using the Mann–Whitney test. For the sample calculation, a large effect size of 0.90, estimated in a previous study by Fregratto et al. (2021) [9]; a margin of error of type 1 (α) of 5%; a study power of 80%; and for the one-tailed test were used. Considering a possible sample loss, 20 sample elements were randomly included by groups. The calculation of sample size and effect size was performed using the G*Power software, version 3.1.9.2 (Franz Faul, Universität Kiel, Kiel, Germany).

As a criterion for the non-inclusion of participants, we used situations such as when physical conditions prevented, for the purposes of clinical analysis, the collection of material and teeth missing from the oral cavity or teeth that were in incomplete eruption (primary dentition: 51, 71, 55, 65, 75, or 85; permanent dentition: 11, 31, 16, 26, 36, or 46). In addition to these factors, participants taking antibiotics at the time of the trial were also excluded.

Oral hygiene assessment and saliva collection were performed by a pediatric dentist with 7 years of experience as well as by a nurse. Both professionals were trained in saliva collection. After seating the participants in a Unik dental chair (Kavo^®^, Joinville, Brazil), or, in some cases, in the wheelchair itself, a clinical assessment of oral hygiene was performed, with subsequent saliva collection. This collection was performed by the dentist wearing personal protective equipment (PPE). As materials, a mouth mirror, mouth swab, disposable plastic suction device, exploration probe, triple syringe, and dental reflector were used. All these precautions were taken to avoid bias.

### 2.4. Oral Hygiene Index—Simplified (OHI-S)

**Figure 1:**
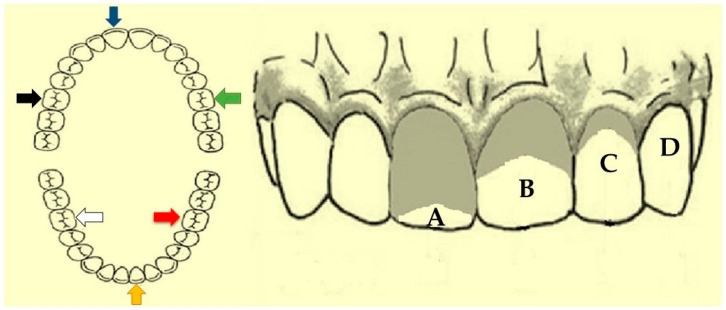
Oral Hygiene Index—Simplified (OHI-S), Malmö University, Sweden. Buccal surface teeth 11 (blue arrow), buccal surface teeth 16 (black arrow), buccal surface teeth 26 (green arrow), buccal surface teeth 31 (yellow arrow), lingual surface teeth 46 (white arrow), and lingual surface teeth 36 (red arrow). Bacterial plaque index: (A) level 3, abundant presence of plaque covering more than 2/3 of the surface; (B) level 2, presence of plaque in the cervical and middle thirds; (C) level 1: presence of plaque in the cervical 1/3 or up to 3 small, isolated collections in other regions; (D) level 0: no plaque. Adapted from Fregatto et al. (2021) [9].

For the oral hygiene assessment, the dental plaque and calculus deposits were quantified using the OHI-S proposed by Greene and Vermillion (1964) [22]. In permanent dentition, the buccal surfaces of teeth 11 (upper right central incisor), 31 (lower left central incisor), 16 (upper right first molar), and 26 (upper left first molar) and lingual surfaces of teeth 36 (lower left first molar) and 46 (lower right first molar) were examined. In deciduous or mixed dentition, the buccal surfaces of teeth 51 (upper right central incisor), 71 (lower left central incisor), 55 (upper right first molar), and 65 (upper left first molar) and lingual surfaces of teeth 75 (lower left first molar) and 85 (lower right first molar) were examined at levels zero to three for plaque and calculus, and the sum of both could reach six. When an index tooth was missing, another tooth from the same dental arch and quadrant was examined. Only fully erupted teeth were selected (Figure 1) [9].

### 2.5. Salivary pH

Salivary pH was analyzed using pH indicator papers (MQuant^®^ Merck, Darmstadt, Germany) with a pH scale from 0 to 14 and graduations of 1 pH, using the colorimetric method. The pH indicator paper was placed in contact with the patient’s right buccal mucosa for 20 s and was subsequently removed. The color change was compared with the scale.

The pH scale had been calibrated through repeated examinations prior to this study. All pH readings were obtained by the same operator under the same lighting conditions, i.e., the light from the reflector attached to the dental chair, as described previously [23].

### 2.6. Salivary Flow

For saliva collection, the participants were instructed not to ingest drinks or food and brush their teeth 1 h before collection, as described previously [24]. For pediatric patients, the caregivers/guardians were instructed to ensure the same. Salivary samples were collected between 8:00 and 10:00 a.m. Sterile compact rolled cotton from the Sarstedt Salivette^®^ tube (Sarstedt, Beaumont Leys, UK) was placed for 1 min in the sublingual region of the patient using sterile metal clinical tweezers until it was completely saturated by saliva; the cotton was subsequently returned to the tube. The flasks were centrifuged, and the saliva settled in the lower compartment of the flask and was free of residues and measures in mL/min.

### 2.7. Saliva Collection

The patients were instructed to rinse their mouths with 100 mL of water, following which the saliva was scraped using circular movements that were performed approximately 30 times on the buccal mucosa with the help of sterile swabs. The swabs were trimmed and placed in 2 mL microtubes containing gel. The samples were stored in a refrigerator at 4 °C for up to 7 days before genomic DNA extraction.

### 2.8. Bacterial Analysis

The salivary samples that were obtained with cotton swabs were tested for *Porphyromonas gingivalis*, *Tanerella forsythia*, and *Treponema denticola*. The mRNA expression was evaluated through polymerase chain reaction (PCR). The analysis of bacteria was performed following the same technique used by the group in the study by Fregatto et al. (2021) [9] as follows:

For DNA extraction, a commercial DNA isolation kit was used (Puregene^®^, Gentra Systems, Minneapolis, MN, USA). In the microtubes containing the swabs, 300 µL of lysis solution was added, followed by 1.5 µL of proteinase K (20 mg/mL) and 100 µL of precipitation solution. Subsequently, 300 µL of 100% isopropanol and 0.5 µL of glycogen (20 mg/mL) were added, and the tubes were centrifuged at 1500 rpm for 3 min. Supernatants were discarded, and the tubes were inverted onto an absorbent paper. Subsequently, 300 µL of 70% ethanol was used to wash the DNA. The tubes were left open for 15 min to help evaporate the residual ethanol, and the DNA was dissolved in 20 µL of a DNA eluting solution [9]. 

The electrophoresis of the DNA samples obtained was carried out in a 1.5% agarose gel with tris/boric acid/ethylenediamine tetra-acetic acid (TBE, 0.001 M, pH 8.0), which contained 0.5 μg/mL ethidium bromide gel and was visualized on a Hoefer transilluminator (Macro Vue UV-20 Hoefer^®^, San Francisco, CA, USA) in order to verify the integrity. The concentration of the DNA samples was measured at 260 nm using a spectrophotometer (Ultrospec III^®^, Pharmacia LKB Biochrom, Cambridge, UK). A 260/280 ratio of ~1.8 was used to characterize the purity of the material. The samples were stored at 4 °C until use [9].

The constitutively expressed glyceraldehyde 3-phosphate dehydrogenase (*GAPDH*) gene was amplified as an internal control for the reactions to confirm whether or not the DNA extraction process was successful. The Invitrogen protocol was adopted as follows:

A sterile tube was added with 2.5 µL of 10× PCR buffer, 0.75 µL of MgCl2 (50 mM), 0.5 µL of dNTP mix (10 mM), 1.0 µL of oligonucleotide primer forward (2 mM), 1.0 µL of oligonucleotide primer reverse (2 mM), 0.5 µL of the DNA sample, 0.2 µL of Taq DNA polymerase (5 IU/µL), and distilled and autoclaved water until a final volume of 25 µL was achieved. The reaction was performed in a Perkin Elmer GeneAmp PCR System 2400 thermal cycler (Perkin Elmer Corporation^®^, Waltham, MA, USA) under the following conditions: denaturation at 94 °C for 45 s, annealing at 60 °C for 45 s, extension at 70 °C for 1 min, and final extension at 70 °C for 15 min for a period of 25 cycles [9].

The FIREpol protocol was adopted to amplify the *Porphiromonas gingivalis, Tannerella forsythia*, and *Treponema denticola* strains using PCR. In a sterile tube, 4 µL of 5× Master Mix, 0.6 µL of forward primer oligonucleotide (10 µM), 0.6 µL for reverse primer oligonucleotide (10 µM), 1.0 µL of the DNA sample, and distilled and autoclaved water were added until a final volume of 20 µL was achieved. This reaction was also performed in a Perkin Elmer GeneAmp PCR System 2400 thermal cycler (Perkin Elmer Corporation^®^, Waltham, MA, USA) under the following conditions: denaturation at 95 °C for 30 s, annealing at 59 °C for 30 s, extension at 72 °C for 1 min, and final extension at 72 °C for 5 min [9].

**Figure 2:**
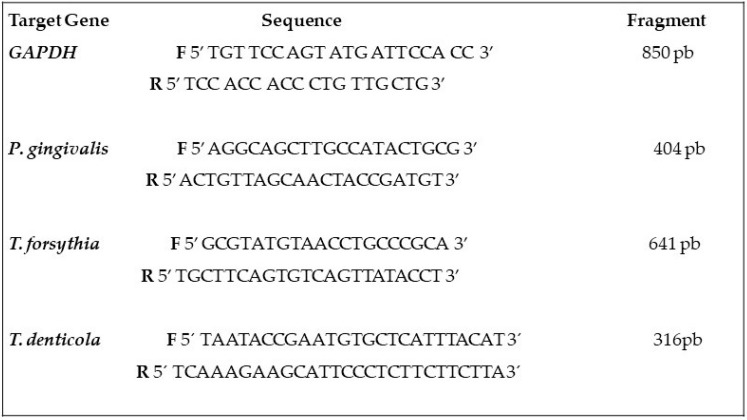
Sequences of oligonucleotide primers used for the amplification of *GAPDH* and bacteria *Porphiromonas gingivalis*, *Tannerella forsythia e Treponema denticola.* bp: base pairs; *GAPDH*: glyceraldehyde 3-phosphate dehydrogenase.

Figure 2 presents the oligonucleotide primer sequences used to amplify the *GAPDH* and specific bacteria identified by Dalpke et al., 2006 [25].

The amplified DNA samples were subjected to electrophoresis on 1.5% agarose gel in a TBE containing ethidium bromide at a concentration of 1.0 µg/mL of gel and visualized on a Hoefer transilluminator (Macro Vue UV-20) to verify the expression of the analyzed genes.

### 2.9. Statistical Analysis

The Kolmogorov–Smirnov test was performed for normality testing at 5% probability. For non-normally distributed data, non-parametric tests were performed. The Mann–Whitney test was performed to compare the groups in terms of their oral hygiene and salivary pH and flow status. Data are expressed as medians (interquartile range). The chi-square test was performed to compare the bacterial flora. The relationship between the dependent variables with age and gender was analyzed using Spearman’s non-parametric correlation test (ρ), to select variables for regression. To analyze the effect of the group on the dependent variables, controlling the effect of gender and age, a multiple linear regression was performed using the Enter method. Linear R^2^ was used to analyze the percentage of variation in the dependent variable explained by the variation in the independent variables of the model. A probability level of 5% was used for both tests. All statistical analyses were performed using the R software package (R Core Team 2020, Vienna, Austria).

## 3. Results

The OHI-S score was poorer in group I than in group II (*p* < 0.05). The Plaque Index score did not differ significantly between the two groups (*p* > 0.05). The Calculus Index score was poorer in group I than in group II (*p* < 0.05; Table 1).

The salivary pH was higher in group I than in group II (*p* < 0.05). The salivary flow did not differ significantly between the two groups (*p* > 0.05; Table 2). One patient was excluded from the evaluation of bacterial flora because the genetic material could not be obtained owing to a technical error.

Table 3 shows the bacterial flora in groups I and II. *Porphyromonas gingivalis* and *Tannerella forsythia* were found in 24 patients, including 14 in group I and 10 in group II. *Treponema denticola* was found in 19 patients, including ten in group I and nine in group II. There was no association between the presence of bacterial flora among the two groups (*p* > 0.05).

The confounding variables of sex and age were analyzed to assess their impact on the dependent variables. Comparing the age between the groups using the Mann–Whitney test, a significant difference (*p*-value = 0.011) was observed for the median (in-interquartile range), with values of 10.0 (7.5) years for group I and 5.0 (4.0) years for group II. Regarding sex, the groups showed similar frequency distribution, without statistically significant differences. 

To explore the effect of gender and age on dependent variables, Spearman’s correlation analysis (ρ) was performed. Spearman’s correlation analysis was used only to identify variables with significant correlation for regression analysis. The results of the correlation analysis were not presented in tables but are described below. There was a positive and significant correlation between age and oral hygiene (ρ = 0.346; *p*-value = 0.028) and calculus (ρ = 0.328; *p*-value = 0.038). As for gender, a significant and negative correlation was observed between females and salivary pH (ρ = 0.312; *p*-value = 0.050) and salivary flow (ρ = 0.391; *p*-value = 0.014).

To analyze the effect of the group on the dependent variables, controlling the effect of sex and age, a multiple linear regression was performed (Table 4). For oral hygiene, a significant effect of the model was observed (*p*-value < 0.001) and the R indicates that the variation in the variables gastrostomy (*p*-value < 0.001) and age (*p*-value = 0.227) explains 38.2% of the variation in oral hygiene. The regression model indicates that the presence of gastrostomy is related to increased oral hygiene. For the calculation, a significant effect of the model was observed (*p*-value < 0.001) and the R^2^ indicates that the variation in the variables gastrostomy (*p*-value < 0.001) and age (*p*-value = 0.808) explain 60.4% of the variation in oral hygiene. The regression model indicates that the presence of calculus is related to the increase in calculus. For salivary pH, a significant effect of the model was observed (*p*-value < 0.001) and the R^2^ indicates that the variation in the variables gastrostomy (*p*-value < 0.001) and sex (*p*-value = 0.094) explains 49.2% of the variation in oral hygiene. The regression model indicates that the presence of gastrostomy is related to the increase in salivary pH. For salivary flow, no significant effect of the model was observed (*p*-value = 0.092) but a significant gender effect was verified (*p*-value—0.044) with a reduction in females (Table 4).

## 4. Discussion

The study results confirmed our hypothesis that children and young people with neurological impairment that are fed via an alternative feeding route have a poorer oral hygiene when compared to those fed orally; this finding is consistent with previous studies showing that gastrostomy negatively affects oral hygiene [13,26,27,28,29,30]. The objective of providing an alternative feeding route is to improve the health of children and young adults with neurological impairment and dysphagia. Therefore, the manner in which this alternative route worsens oral health should be understood.

The increased dental calculus deposition indicates the worsened oral hygiene in children and young adults with neurological impairment and dysphagia fed via an alternative feeding route. Moreover, it is associated with the absence of food in the oral cavity, since food plays a role in maintaining the composition and pH of saliva [31,32]. Additionally, in children fed via gastrostomy, the intraoral mechanical forces of mastication are partially or completely eliminated, allowing the depositions of pathogenic bacterial plaque and dental calculus [33]. 

In this study, children and young people with neurological impairment fed via gastrostomy had a more basic salivary pH compared to those fed orally, consistent with previous study findings. In a previous study involving 25 patients with cerebral palsy that included 15 patients fed via gastrostomy and 10 patients fed orally, those fed via gastrostomy showed a more basic pH compared to those fed orally. The salivary parameters of patients with cerebral palsy fed via gastrostomy were significantly increased compared to those fed orally. This finding may explain the excessive dental calculus deposition in patients fed exclusively via gastrostomy [34].

In another study, a more basic salivary pH was identified in patients fed via an alternative route because of the absence of food in the oral cavity. When the salivary pH is above 5.5, plaque becomes supersaturated and deposits minerals; this occurs in patients fed via an alternative route because their plaque is not exposed to fermentable carbohydrates [35].

The relatively basic pH in patients with neurological impairment fed via gastrostomy because of the absence of food in the oral cavity can also be explained through the physiology of saliva, which has organic and inorganic components. The inorganic components modulate the demineralization and remineralization, while the organic components, i.e., nitrogen products (such as ammonia and urea), bicarbonates, and phosphates, modulate the buffering capacity and pH of saliva [36].

In this study, the salivary flow did not differ between children and young people with neurological impairment fed via gastrostomy and the oral route. In both groups, salivary flow was within the normal ranges (adults, 0.25–0.35 mL/min; children, <1.6 mL/min) [37,38].

Evidence is limited regarding the salivary flow in children and young people with neurological impairment fed via an alternative route. Most studies were focused on quantifying sialorrhea and treatment planning [7,39,40,41]. Few studies indicated a reduced salivary flow rate and buffering capacity of saliva, suggesting salivary gland involvement in spastic cerebral palsy. Moreover, sialorrhea in these patients did not result from excess saliva production but from oropharyngeal dysphagia, sensory alterations, and oromotor dysfunctions. Notably, more studies should be conducted on the possible autonomic dysfunctions, hypohydration, and/or impairment of salivary gland function in patients with neurological impairments [16,42].

Consistent with previous studies, salivary flow did not increase beyond normal values in the present study. No previous study has investigated the difference in the oral hygiene status of children and young people with neurological involvement with and without gastrostomy. However, in this study, salivary flow did not differ between the two groups, suggesting that salivary flow is not altered by the use of an alternative feeding route.

In this study, *Porphyromonas gingivalis*, *Tanerella forsythia*, and *Treponema denticola* were present in the oral cavity of children and young people with neurological impairment, with no significant difference between those fed via gastrostomy and the oral route. Literature indicates that these three bacteria are usually present in the oral cavity of children and adults with or without neurological impairment [43,44]. These species constitute the “red complex” and are periodontopathogenic bacteria, causing periodontitis and increasing its severity [45,46]. Moreover, in the presence of these three bacterial species, the periodontal health status is worsened [47]. A previous study indicated that *Porphyromonas gingivalis* is rarely found in the saliva of periodontally healthy children and young adults but frequently found in the saliva of adult patients with periodontitis [48].

Although the correlation analysis indicated a significant correlation between age and sex on oral hygiene, calculus, salivary pH, and salivary flow, the correlation coefficient values indicate a moderate to low correlation. In addition, the effect of gender and age was not confirmed for oral hygiene, calculus, and salivary pH in the regression analysis, which showed the main effect of the group. It is also worth noting, considering the values of linear R^2^, that gastrostomy has a great impact on the variables oral hygiene, calculus, and saliva pH after controlling for the covariates age and gender.

We hypothesized that children with neurological impairment fed via gastrostomy present with poor oral hygiene and, consequently, the greater accumulation of these bacteria due to the increased dental calculus deposition. However, this hypothesis was not confirmed, possibly because of the small sample size and presence of these species in the healthy population. A 1997 study analyzed the oral microbiota in children with and without gastrostomy and found no difference in the presence of the analyzed bacteria. The present study suggested that oral consumption of food is not a determining factor for the presence of oral microbiota in children [25].

Scientific evidence on the analysis of these three bacterial species in the oral cavity of children and young people with neurological impairment fed via an alternative feeding route is scarce [49]. A 2020 systematic review and meta-analysis showed that studies on oral health and cerebral palsy analyzed the oral hygiene, malocclusion, and caries [50] in the corresponding population. A recent study analyzed the oral health in children with neurological impairments, such as cerebral palsy, Down’s syndrome, and autism; however, they focused on oral hygiene and caries and not on oral microflora [29]. Most studies have analyzed the prevalence of the three bacterial species in the oral cavity of children with neurological impairment, such as Down’s syndrome, and reported that the most frequent bacterial combination is the red complex [51,52,53].

The scientific evidence on the salivary flow and analysis of *Porphyromonas gingivalis*, *Tanerella forsythia*, and *Treponema denticola* in children and young people with neurological disorders fed via an alternative feeding route is limited. However, the present study could serve as a basis for introducing preventive measures for the care of oral hygiene in children and young adults with neurological impairment fed via an alternative feeding route, providing a reference for the analysis of poor oral hygiene, greater dental calculus deposition, and higher salivary pH due to the absence of food in the oral cavity in children and young adults with neurological impairment fed via gastrostomy. Our findings suggest that food should be introduced into the oral cavity, even for salivary stimulation, as it would help maintain the composition and pH of saliva and, consequently, the oral health of children and young adults with neurological impairment fed via an alternative feeding route.

However, this study has a few limitations. First, children and young people without neurological involvement were not included. Second, the sample size was small. Therefore, future large-scale studies involving healthy children and young adults as control are warranted. Although the sample may be considered small, the calculation of the sample size indicates that the study has sufficient sample elements for the purposes of the study. However, for the analysis of bacteria, it is believed that future studies with larger samples are necessary. However, it is worth noting that this population has limitations that make it difficult to include many participants in the sample, which can also contribute to a lower sample homogeneity and an increase in confounding factors.

Within these limitations, this study could contribute to the improvement in oral care of this population, including routine multidisciplinary dental care, involving physicians, speech therapists, nurses, dentists, and nutritionists, to maximize the care and quality of life of both children and young adults with neurological impairment and their families.

## 5. Conclusions

This study compared oral hygiene, salivary pH, salivary flow, and the oral microbiota of patients with neurological impairment and oropharyngeal dysphagia fed via gastrostomy or orally. Children and young people with neurological impairment and oropharyngeal dysphagia fed via gastrostomy presented with poor oral hygiene, greater dental calculus deposition, and higher salivary pH. As for bacterial species, both children and young adults fed via gastrostomy and via the oral route presented with the presence of *Porphyromonas gingivalis*, *Tanerella forsythia*, and *Treponema denticola* in the oral cavity.

## Figures and Tables

**Table 1:** Values of medians and interquartile range of the oral hygiene index, plaque and calculus in relation to groups I and II, and the total number of children and young people.

	Group I	Group II	Total	*p*-Value *
	Median (Interquartile)	Median (Interquartile)	Median (Interquartile)	
Oral Hygiene (level)	4.00 (1.68)	2.00 (0.93)	2.50 (2.4)	0.0003
Plaque	2.10 (1.08)	1.80 (0.5)	2.00 (0.7)	0.2939
Calculus	2.00 (0.85)	0.00 (0.08)	0.50 (2)	0.0000

Group I: 20 children and young people fed via gastrostomy; Group II: 20 children and young people fed orally, Level: 0–6. * Mann–Whitney Test. Values were calculated medians (interquartile).

**Table 2:** Values of medians and interquartile range of pH and salivary flow in relation to groups I and II and the total number of children and young people.

	Group I	Group II	Total	*p*-Value *
	Median (Interquartile)	Median (Interquartile)	Median (Interquartile)	
Salivary pH	7.50 (1.0)	6.00 (0.62)	6.70 (1.5)	0.0000
Salivary flow	0.80 (0.5)	0.70 (0.72)	0.70 (0.7)	0.1168

Group I: 20 children and young people fed via gastrostomy; Group II: 20 children and young people fed orally. * Mann–Whitney Test. Values were calculated medians (interquartile).

**Table 3:** Absolute and relative frequency (%) of individuals with the presence and absence of the bacteria *Porphyromonas gingivalis*, *Tannerella forsythia*, and *Treponema denticola* in groups I and II.

	*Porphyromonas gingivalis*		*Tannerella forsythia*		*Treponema denticola*	
	No	Yes	No	Yes	No	Yes
Group I (%)	5 (26.3)	14 (73.7)	5 (26.3)	14 (73.7)	9 (47.4)	10 (52.6)
Group II (%)	10 (50)	10 (50)	10 (50)	10 (50)	11 (55)	9 (45)
Total (%)	15 (38.5)	24 (61.5)	15 (38.5)	24 (61.5)	20 (51.3)	19 (48.7)
*p*-value *	0.2339		0.2339		0.8759	

Group I: 20 children and young people fed via gastrostomy; Group II: 20 children and young people fed orally. * indicates the chi-square test. Values were calculated absolute and relative frequency (%).

**Table 4:** Multiple linear regression analysis to verify the effect of age and sex covariates on the variables that showed significant correlation.

Variables		Β	95% CI		*p*-Value *	Model	
Dependent	Independent		Lower Bound	Upper Bound		*p*-Value	R^2^
Oral Hygiene	Gastrostomy (1 = yes; 2 = no)	−1.540	−2.353	−0.727	<0.001	<0.001	0.382
	Age (years)	0.042	−0.035	0.120	0.277		
Calculus	Gastrostomy (1 = yes; 2 = no)	−1.579	−2.049	−1.109	<0.001	<0.001	0.604
	Age (years)	0.005	−0.040	0.050	0.818		
Salivary pH	Gastrostomy (1 = yes; 2 = no)	−1.197	−1.656	−0.737	<0.001	<0.001	0.492
	Gender (1 = male; 2 = female)	−0.392	−0.854	0.070	0.094		
Salivary flow	Gastrostomy (1 = yes; 2 = no)	−0.064	−0.334	0.205	0.630	0.092	0.124
	Gender (1 = male; 2 = female)	−0.277	−0.547	−0.007	0.044 *		

Note. Regression coefficient (β); 95% confidence interval (95%CI); coefficient of determination (R^2^). * Multiple linear regression test.

## Data Availability

The data presented in this article are available from the corresponding author upon request.
